# Economic Activity and Trends in Ambient Air Pollution

**DOI:** 10.1289/ehp.0901145

**Published:** 2010-01-04

**Authors:** Mary E. Davis, Francine Laden, Jaime E. Hart, Eric Garshick, Thomas J. Smith

**Affiliations:** 1 Department of Urban and Environmental Policy and Planning, Tufts University, Medford, Massachusetts, USA; 2 Exposure, Epidemiology and Risk Program, Department of Environmental Health, Harvard School of Public Health, Boston, Massachusetts, USA; 3 Channing Laboratory, Brigham and Women’s Hospital and Harvard Medical School, Boston, Massachusetts, USA; 4 Department of Epidemiology, Harvard School of Public Health, Boston, Massachusetts, USA; 5 Pulmonary and Critical Care Medicine Section, Medical Service, VA Boston Healthcare System, West Roxbury, Massachusetts, USA

**Keywords:** air pollution, business cycle, chronic disease, diesel, economy, epidemiology, traffic exposure, trucking industry

## Abstract

**Background:**

One challenge in assessing the health effects of human exposure to air pollution in epidemiologic studies is the lack of widespread historical air pollutant monitoring data with which to characterize past exposure levels.

**Objectives:**

Given the availability of long-term economic data, we relate economic activity levels to patterns in vehicle-related particulate matter (PM) over a 30-year period in New Jersey, USA, to provide insight into potential historical surrogate markers of air pollution.

**Methods:**

We used statewide unemployment and county-level trucking industry characteristics to estimate historical coefficient of haze (COH), a marker of vehicle-related PM predominantly from diesel exhaust. A total of 5,920 observations were included across 25 different locations in New Jersey between 1971 and 2003.

**Results:**

A mixed-modeling approach was employed to estimate the impact of economic indicators on measured COH. The model explained approximately 50% of the variability in COH as estimated by the overall *R*^2^ value. Peaks and lows in unemployment tracked negatively with similar extremes in COH, whereas employment in the trucking industry was positively associated with COH. Federal air quality regulations also played a large and significant role in reducing COH levels over the study period.

**Conclusions:**

This new approach outlines an alternative method to reconstruct historical exposures that may greatly aid epidemiologic research on specific causes of health effects from urban air pollution. Economic activity data provide a potential surrogate marker of changes in exposure levels over time in the absence of direct monitoring data for chronic disease studies, but more research in this area is needed.

The lack of adequate historical air pollution data is an important problem for exposure classification in epidemiologic studies of chronic disease. The temporal and spatial availability of pollution monitoring data becomes scarcer the farther back in time exposure begins. Thus, obtaining unbiased estimates of lifetime dose related to long-term exposures is difficult. The studies that have access to quantitative exposure data are typically limited to current or recent exposures for which air pollution monitoring data are readily available, with little or no information available on past levels (Yanosky et al. 2009a, 2009b). Often, point estimates or averages of current exposure are assumed to be representative of lifetime exposures or related in some linear fashion to earlier exposures, with higher levels generally assumed in the past (for a review of long-term air pollution studies, see Pope and Dockery 2006). This type of extrapolation does not address the temporal or spatial variability in long-term exposures, such as changes over time, across cities and regions, or the urban–rural gradient. It also does not account for the fact that changes in exposure patterns over time are not uniform across spatial locations, that is, temporal patterns in chronic exposure will vary from place to place.

Given the relative paucity of direct air pollution monitoring data, it is useful to define alternative variables that represent surrogates of exposure that are more widely available historically. This paper explores one potential source of predictive data, economic activity, and its association with the coefficient of haze (COH). COH is a historically available marker of combustion soot from vehicles, especially diesel exhaust, as well as from oil furnaces and some industrial processes. We hypothesize that local economic activity is correlated with exposure conditions for air pollutants and that it is strongly related to pollution output at the local level. Although the regional transport of pollutants will also play an important role, this paper concentrates more specifically on the impact of local sources by investigating city- and county-level data from a single state, New Jersey, USA. This work may be particularly pertinent to occupational health studies, where business activity is more strongly associated with worker exposures.

## Methods

### Historical exposure data

The COH represents an early air pollution monitoring technique designed to assess compliance with total suspended particulate (TSP) standards [U.S. Environmental Protection Agency (EPA) 1983]. It is derived from a filter-tape sampling method where a device clamps on the tape and draws air through at a fixed rate for 2 hr, then unclamps, rolls the tape forward, and reclamps for another 2-hr sample. This leaves a series of sequential spots. During each sample, a light shines through the tape to a photocell measuring the optical density of the spot, which is reported in COH units. This monitor was developed in 1953 and widely employed in the 1960s and 1970s (U.S. EPA 1983).

Although it was initially designed as a marker of TSP, the COH measurement method is more strongly predictive of smaller respirable particles in particulate matter < 1 μm in diameter (PM_1_), including black carbon (BC) (*R*^2^ = 0.99; Allen et al. 1999) and elemental carbon (EC) (*R*^2^ = 0.94; Cass et al. 1984). BC and EC are highly correlated (*R*^2^ = 0.93; Allen et al. 1999) and are frequently used as a marker of vehicle exhaust particulate, especially from diesel sources [National Institute for Occupational Safety and Health (NIOSH) 1998; Smith et al. 2006; Steenland et al. 1990; Zaebst et al. 1991]. The COH measurement indicates all of the sources of black and dark-colored PM (Allen et al. 1999; Cass et al. 1984), so there is some error in its application as an index of diesel soot particles depending on the preponderance of other local soot sources, such as oil heating systems. Kirchstetter et al. (2008) used archived COH data from California to assess historical trends in diesel vehicle emissions in the San Francisco Bay area. To the knowledge of the authors, this is the first attempt to analyze COH trends on the East Coast.

Although much of the archived COH data across the country have been lost because of changes in data storage methods over time, an extensive 30-year data set of monthly COH levels from 1971 to 2003 for 25 locations in New Jersey was obtained from A. Mikula at the New Jersey Department of Environmental Protection (personal communication, 2009). These locations cover all but three (Cape May, Cumberland, and Sussex) of the 21 counties in the state, which range from highly urban to suburban and rural. Table 1 provides a list of the locations and years covered by these data.

This COH data set is well suited to the task of outlining the long-term impact of the business cycle on air pollution exposure from primarily diesel-related sources over time. In this context, home heating with oil will not be as sensitive to economic variations, and heating will not affect summer air pollution levels. Although other particle-phase air pollutants have been monitored nationally over different segments of this time frame (TSP 1971–1987; PM_10_ 1980–current; and PM_2.5_ 1997–current), none provide the temporal and spatial resolution of the COH data for this region. In addition, an analogous data set could not be reconstructed from the mixed-particle observations of various size ranges, as the particles come from different sources and are often not highly correlated. The completeness of the COH data enables a more thorough investigation of the long-term economic link to human health across time.

### Economic and regulatory data

State-level unemployment rates are readily available online from the U.S. Census Bureau starting in 1970 (U.S. Census Bureau 2009a). They represent the percentage of the state population designated as unemployed during a given year. Because the majority of diesel vehicles in the United States are heavy-duty trucks, we also focused on trucking sector employment as a surrogate for the number of operating diesel vehicles in a given year. Annual county-level data on the number of trucking employees per square mile are readily available online from the U.S. Census Bureau starting in 1964 (U.S. Census Bureau 2009b). We also control for weather-related factors including temperature and precipitation. Summary statistics for these model covariates are provided in Table 2.

We further identified five regulatory periods, defined by the major federal regulations that impacted vehicle emissions for diesel as well as gasoline engines (Table 3). A lag period of 1 year was incorporated into these variables to allow the impact of the regulations to set in, although longer lag periods were also tested in the model. These regulatory periods of interest were the Clean Air Act (1970) and smoke standards for diesel engines, the 1978 passage of the Corporate Average Fuel Economy Standards (Energy Policy Conservation Act 1975), and regulations limiting PM emissions from diesel engines in 1988, 1994, and 1996. (U.S. EPA 1997).

### Statistical modeling approach

The location data were not uniformly distributed over time; for this reason a mixed-modeling approach was applied using the methods developed by Baltagi and Wu (1999). This approach accounts for unequally spaced panel (longitudinal) data using generalized least squares estimation procedures and incorporates a first-order autoregressive error term [AR(1)]. The “xtregar” command in Intercooled STATA Version 10.0 (College Station, TX) was used to estimate the following equation:


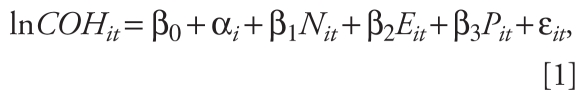


where ɛ*_it_* = ρɛ*_it_* − 1 + η*_it_*, α*_i_* = random location effect, *N* = weather variables, *E* = economic variables, *P* = regulatory periods, *i* = location, and *t* = month/year.

The COH data were approximately log normally distributed and were log transformed. α*_i_* represents a random-location effect that controls for differences across the 25 different monitoring locations, and the error term ɛ*_it_* is a function of both the previous period ɛ*_it_*−1 (where ρ is the correlation across time periods) and a random component (η_i_). *N* includes temperature and precipitation, whereas *E* incorporates both statewide unemployment as well as county-specific trucking employment. Finally, the regulatory periods were included as dummy variables in P to control for their impact over time. For estimation purposes, the initial regulatory period was excluded, and all later periods were judged against the first period.

## Results

**Figure 1:**
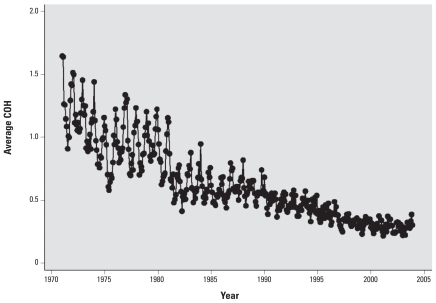
COH trend in New Jersey (1971–2003).

Figure 1 provides a graphical display of the monthly COH trends in New Jersey. We found a strong downward trend in COH over time, with an approximate 75% decline between 1971 and 2003. A relatively large decline was observed in the 1970s, followed by a leveling off during the 1980s, and another steadily decreasing trend in the 1990s. The variability in monthly location-specific COH levels around the annual mean was measured by the annual coefficient of variation [COV; ratio of the standard deviation and the annual mean (σ/μ)]. Overall, the COV averaged around 45% across the 30-year period, with a slight increasing trend in the later years. Therefore, although the graph shows a decline in absolute variability and noise over time, the relative variability (variation around the annual mean) was fairly stable.

**Figure 2:**
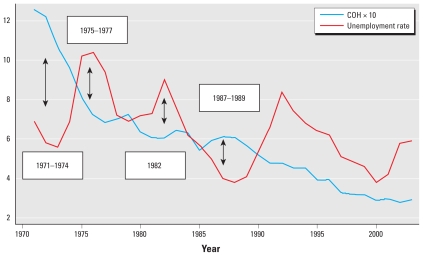
COH and unemployment in New Jersey. The *y*-axis represents scaled COH (COH × 10) super-imposed with a measure of the percentage of the unemployment rate. Arrows indiate the relationship between unemployment rates and COH.

When we matched data on statewide unemployment and county-level trucking employment, a visible pattern emerged to suggest a relationship between these economic indicators and COH levels. In Figure 2, short-term highs and lows in state-level unemployment in New Jersey were negatively associated with the highs and lows in statewide annual average COH levels. Beginning in the initial period (1971–1974), low unemployment rates corresponded to relatively high levels of COH. As the unemployment rate increased by approximately two thirds over the following years (1975–1977), COH levels had a short-term drop of approximately 35% during this same period. Extremes in the unemployment rates during 1982 and 1987–1989 displayed a similar relationship between COH and unemployment over the short term, with high levels of unemployment corresponding to decreased levels of COH and low unemployment corresponding to increased COH. However, this relationship appeared to break down beginning in the early 1990s.

**Figure 3:**
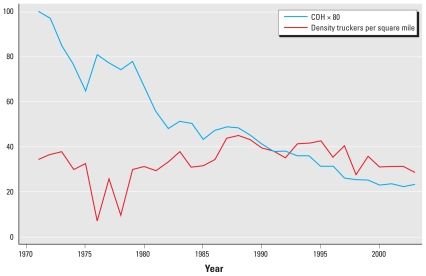
COH and trucking employment in New Jersey. The *y*-axis represents scaled COH (COH × 80) superimposed with a measure of the density of trucking employees per square mile.

In Figure 3, a similar pattern was distinguishable between COH and trucking employment, with the sharp decline in COH levels occurring around the same period of abnormally low trucking employment. In the 1980s, this relationship continued with a strong positive association between trucking employment and COH, that is, dips in COH were associated with similar declines in trucking employment and vice versa.

**Figure 4:**
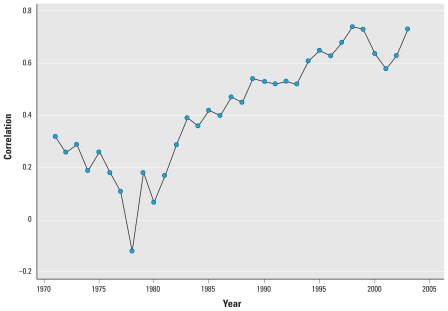
Annual correlation coefficient of COH (log transformed) and trucking employment density.

The Pearson correlation coefficients in Table 4 provided further quantitative evidence of these trends. To be consistent with the later statistical model, the correlation between employment indicators and COH (log transformed) were calculated within each regulatory period, although similar results were found using different time scales, for example, correlations within 5-year increments. The relationship between unemployment and COH was relatively strong during the 1970s. However, this relationship broke down over later periods. The opposite was true for trucking employment per square mile, where the relationship with COH became progressively stronger through time. When we looked more closely at the yearly correlation coefficients for COH and trucking employment density displayed in Figure 4, this relationship showed consistently positive correlations across all years except 1978 (*r* = −0.12).

The results from the random-effects model are presented in Table 5. A total of 5,920 observations were included across 25 different locations, and the model explained approximately 50% of the variability in COH as estimated by the overall *R*^2^ value. All coefficients had the appropriate sign, with the expected winter heating effect at colder temperatures (lower temperatures are predictive of higher COH levels), and the negative impact of precipitation (effectively cleaning particles out of the air). Unemployment had the expected negative impact on COH. The regression results estimated that a 1-SD increase above the mean unemployment rate (from 6.5 to 8.3%) would lead to a 5.8% decline in COH levels. The density of trucking employees was also in line with expectations and showed a positive and significant coefficient. A 1-SD increase in the density of trucking employment above average levels (from 32.4 to 102.1 employees per square mile) would subsequently increase expected COH levels by 7.8%.

The regulatory periods of interest played a large and significant role in predicting COH levels. Each regulatory period was associated with a significant decline in COH against the initial baseline period (1971–1978), although the marginal effect of regulations decreased through time (period 2: −29%; period 3: −15.1%; period 4: −14.9%; and period 5: −10.2%). A lag period of 1 year was incorporated into these variables to allow the impact of the regulations to set in, although longer lag periods were also tested in the model. The regression results were robust to the different lag specifications, with similar coefficient effect sizes and significance levels. However, the overall fit declined slightly with increasing time from the initial period of regulation (*R*^2^ = 0.46: 1-year lag; *R*^2^ = 0.43: 2-year lag; *R*^2^ = 0.41: 3-year lag, etc.), suggesting that the 1-year lag provided the best fit for this model. Finally, although the marginal impact of each regulation decreased through time, all had the expected negative and significant coefficient sign, which suggests that federal regulations were ultimately successful at lowering COH ambient concentrations in New Jersey.

Separate random-effects models were also run within each regulatory period, and these results are shown in Table 6. The goal of the additional analyses was to explore the changing relationship between economic indicators and COH over time, as well as isolate the partial effect of the economic variables by estimating the relationship within (as opposed to across) regulatory periods. The *R*^2^ values over the five separate regression models ranged from 0.20 to 0.43. The coefficients mirrored the results discussed earlier with positive and significant coefficients for trucking employment density and negative and significant coefficients on unemployment in the earlier but not later periods.

## Discussion

Economists have long speculated about the impact of business cycles on human health. Most evidence has suggested a counter-intuitive positive relationship between economic downturns and health, that is, people are healthier in bad economic times (Brenner 1971, 1973, 1975, 1979, 1987, 1995; Eyer 1977; Forbes and McGregor 1984; Gerdtham and Johannesson 2005; Gravelle et al. 1981; Johansson 2004; Joyce and Mocan 1983; Laporte 2004; McAvinchey 1988; Neumayer 2004; Ogburn and Thomas 1922; Ruhm 2000, 2003, 2005; Thomas 1927). Economic theory typically predicts a positive association between consumption and income, that is, people consume more at higher income levels. This pattern applies equally to products that are bad for your health, such as dining out, cigarettes, and alcohol. In periods of economic growth, the theory supports an increase in “bad behaviors” and adverse health effects, whereas the opposite would occur during recessionary periods.

In this paper, we present an alternative hypothesis to explain the apparent relationship between cyclical economic activity and human health that suggests that changes in ambient pollution levels are responsible, at least in part, for this apparent link.

Other supportive evidence from the health and economics literature is consistent with the theory that economic conditions impact human health through changes in air pollution exposure. Pope (1989) showed that reductions in the criteria air pollutant PM_10_ after the closure of a major industrial polluter in Utah was associated with health improvements in the local population as measured by the number of hospital admissions for respiratory distress. Clancy et al. (2002) provided evidence that improvements in cardiovascular and respiratory health outcomes were the direct result of a 1990 regulatory decision to ban the sale of coal in Dublin, Ireland, and work by Chay and Greenstone (2003) further linked reductions in infant mortality with the 1981–1982 recession and declining industrial output.

The link between the economic indicators and air pollution has also been noted in the exposure science literature. In a study of ozone levels in Switzerland (Kuebler et al. 2001), concentrations were highly correlated with the European Union gross national product and industrial production growth rates, both indicators of economic activity in the region. Furthermore, a recent study of elemental carbon in the U.S. trucking industry (Davis et al. 2006, 2007) showed that job-related exposure to vehicle exhaust was significantly related to business activity at individual trucking terminals, such as the size of operation and number of employees. These studies provide direct evidence in support of the hypothesis that economic and regulatory activities have a significant impact on exposure to pollution and ultimately human health.

Our results provide evidence of a significant relationship between employment characteristics and COH levels. Peaks and lows in unemployment are negatively associated with similar extremes in COH, and county-level trucking employment levels are positively associated with COH. The relationship between unemployment and COH starts to become less apparent after the oil crises of the 1970s, whereas the opposite is true for the relationship between trucking employment and COH, which strengthens through time. One plausible explanation for the declining impact of unemployment is related to changing patterns of consumer spending over time, and in particular evolving attitudes and price sensitivity toward gasoline prices. Hughes et al. (2008) noted that although a 20% increase in gasoline prices in 1975–1980 resulted in a 6% decline in consumption nationwide, the same relative price increase in 2001–2006 resulted in a more modest 1% decline. This suggests that consumer spending on pollution-generating transportation fuel was more responsive to changing economic conditions in the past, which is reflected by the relatively strong relationship between unemployment and COH in the 1970s. In contrast, the density of trucking employment is more broadly linked to overall economic activity and less susceptible to the price sensitivity specific to gasoline prices, as noted for unemployment. Trucking companies respond quickly to an economic downturn by reducing labor costs, and lower employment levels in the industry are significantly related to economic output and pollution-generating activities. One possible explanation for the growth in the correlation of trucking employment density and COH over time is the declining influence of labor unions. Labor unions were more active during earlier periods, and their advocacy significantly reduces the ability of trucking companies to respond to worsening economic conditions by firing workers.

A strong association was also seen between regulatory policies and COH levels in New Jersey. Federal air-quality regulations were significantly predictive of the sharp decline in COH levels seen in New Jersey over the study period. The early regulations of the 1970s and 1980s had the largest impact on air quality, with the marginal impact of each additional regulatory policy declining through time. However, it is unclear to what extent the marginal effects were attributable to the specific legislation or were influenced by the regulatory inertia created by early regulations. The strength of the association across regulatory periods and COH trends suggests that the federal policies were ultimately successful in their goal of improving air quality and essentially tempered the increasing trend in pollution that otherwise would have resulted from economic growth. These results provide strong evidence to support the inclusion of relevant policy changes in chronic dose estimation for epidemiological studies of environmentally related diseases.

COH declined sharply in New Jersey over the study period—approximately 75% between 1971 and 2003. However, there was a noticeable slowing in the otherwise steady decline in COH that occurred during the 1980s. Kirchstetter et al. (2008) noted a similar leveling out of black carbon concentrations during this time period in San Francisco, California. This may be explained in part by the sporadic changeover to diesel-fueled vehicles in the U.S. trucking fleet during that period. Although long-distance trucks (travel > 100 miles) had been using diesel fuel well before 1970, it was not used extensively in the local intercity delivery trucks until the 1980s (Garshick et al. 2002). The influx of diesel-fueled trucks during this period may have been responsible for the stall in the decreasing trend that otherwise occurred throughout the entire study period.

The significant relationships observed for both measures of employment and COH levels provides some evidence to suggest that economic trends may be useful in historical exposure modeling and chronic dose estimation in epidemiologic studies. Because of the lack of available retrospective exposure data, epidemiologic studies often use currently measured point estimates or exposure averages to recreate lifetime exposures that may occur over many decades. In these cases, economic data may be used to update the exposure averages and incorporate greater spatial and temporal resolution. For example, exposure estimates for individuals within an epidemiologic cohort may be based primarily on an exposure model constructed from available recent exposure data, and these estimates may be adjusted historically (or spatially) using the more readily available historical economic data. This work may be especially useful for updating cumulative exposure estimates in occupational studies, where the link between economic activities as a pollution-generating source is stronger than general population studies. The enhanced spatial and temporal resolution in the cumulative dose estimation would reduce misclassification bias across study subjects, and the application would therefore be more broadly useful for risk assessment and policy purposes.

Further work is needed to validate the trends observed in this study to other regions of the United States. Work is currently underway to replicate this model with a second extensive COH data set from the state of California. This research clearly demonstrates that the relationship between COH and economic trends is not constant through time and that this temporal variability must be accounted for if these data are to be adequately used to update historical exposure models. Although these data are widely available in the United States, their accessibility in other parts of the world is highly country-specific; there are likely to be wide gaps in data availability and error in less-developed countries.

## Conclusions

Nearly half of the variability in COH levels is explained by this parsimonious model using economic activity levels, environmental regulations, and weather characteristics. Therefore, we conclude that this method may potentially be useful for estimating historical exposures to air pollution for chronic epidemiologic studies. However, further research in this area is necessary to fully illuminate the relationship between economic trends and air pollution levels. More specifically, it would be useful to expand on the current mixed-modeling approach to incorporate more site-specific sources around each of the monitoring locations within a land use regression-style framework. In addition, a comparative analysis of the historical COH trends between the East and West Coast of the United States (New Jersey and California) would provide important information on the changing mix of sources across time and geography. Finally, it would be useful to test this relationship for other air pollutants, such as ozone and sulfur dioxide, as well as to incorporate health outcomes data to examine whether this alternative approach provides a stronger link between cumulative exposure and environmentally related chronic diseases.

## Figures and Tables

**Table 1:** Description of COH data in New Jersey.

			County	
Location	County	Years	Population density^a^	Median COH^b^
Ancora State Hospital	Camden	1971–2001	2,326	0.33
Asbury Park	Monmouth	1971–1983	1,346	0.49
Atlantic City	Atlantic	1971–1986	484	0.70
Bayonne	Hudson	1971–1983	12,875	0.92
Burlington	Burlington	1971–2003	560	0.43
Camden (two sites)	Camden	1971–2003	2,325	0.33
East Orange	Essex	1980–1983	6,225	0.64
Elizabeth (two sites)	Union	1971–2003	5,142	0.72
Flemington	Hunterdon	1980–2003	304	0.22
Freehold	Monmouth	1971–2003	1,346	0.49
Hackensack	Bergen	1971–2003	3,860	0.54
Jersey City	Hudson	1971–2003	12,875	0.92
Morristown	Morris	1971–2003	1,052	0.54
Nacote Creek	Atlantic	1979–1983	484	0.70
Newark	Essex	1971–2003	6,226	0.64
Paterson	Passaic	1971–1982	2,682	1.03
Paulsboro	Gloucester	1971–1980	869	0.83
Penns Grove	Salem	1971–1985	197	0.54
Perth Amboy	Middlesex	1971–2003	2,541	0.49
Phillipsburg	Warren	1971–1982	310	0.74
Somerville	Somerset	1971–1982	1,064	0.63
Toms River	Ocean	1971–1998	884	0.50
Trenton	Mercer	1971–1981	1,627	0.68

a Represented by the number of people per square mile in 2000 (U.S. Census Bureau 2009c).

b Data provided by A. Mikula, New Jersey Department of Environmental Protection (personal communication).

**Table 2:** Summary statistics.

Variable	Obs	Mean	Median	SD	Scale	Source
COH	6,232	0.64	0.55	0.40	City/monthly	A. Mikula, personal communication
Temperature (°F)	6,232	52.8	52.7	15.4	State/monthly	NCDC 2009
Precipitation (inches)	6,232	3.9	3.8	1.8	State/monthly	NCDC 2009
Unemployment rate^a^	5,920	6.5	6.4	1.8	State/annual	U.S. Census Bureau 2009a
Trucking employees^b^	6,232	32.4	6.4	69.7	County/annual	U.S. Census Bureau 2009b

Abbreviations: NCDC, National Climatic Data Center; Obs, number of observations.

a State-level unemployment statistics were not available from the U.S. Census Bureau for 1990 and 1998.

b Represented by the number of trucking employees per square mile.

**Table 3:** Regulatory time points.

Time period	Description
1971–1978	1970: U.S. EPA created, first Clean Air Act passed, and smoke standards set for diesel engines^a^
1979–1988	1978: Implementation of Corporate Average Fuel Economy fuel standards^b^
1989–1994	1988: standards for PM emitted from diesel engines strengthened to 0.6 g/bhp-hr^c^
1995–1996	1994: standards for PM emitted from diesel engines (trucks) strengthened to 0.1 g/bhp-hr^c^
1997–2003	1996: standards for PM emitted from diesel engines (buses) strengthened to 0.05 g/bhp-hr^c^

a Clean Air Act (1970).

b Energy Policy Conservation Act (1975).

c U.S. EPA (1997).

**Table 4:** Pearson correlation coefficients for COH^a^ versus employment.

	Regulatory period				
	1 (1971–1978)	2 (1979–1988)	3 (1989–1994)	4 (1995–1996)	5 (1997–2003)
Unemployment rate	−0.27	−0.003	−0.15	−0.02	−0.01
Truck employment per square mile	0.20	0.25	0.54	0.63	0.65

a Log transformed.

**Table 5:** Random effects estimation of COH.

Variable error	Coefficient	SE
Temperature	−0.01**	0.0003
Precipitation	−0.01**	0.002
Unemployment rate	−0.03**	0.01
Truck employment per square mile	0.001*	0.0003
Regulatory period 2^a^	−0.34**	0.02
Regulatory period 3^a^	−0.58**	0.03
Regulatory period 4^a^	−0.89**	0.04
Regulatory period 5^a^	−1.18**	0.03
Constant	0.29**	0.08

Number of observations = 5,920; number of locations = 25; Prob > χ^2^ = 0.000; *R*^2^ overall = 0.45.

a Regulatory periods identified in Table 3.

* *p* < 0.05.

** *p*< 0.01.

**Table 6:** Random effects estimation of COH by regulatory period.

	Regulatory period				
	1 (1971–1978)	2 (1979–1988)	3 (1989–1994)	4 (1995–1996)	5 (1997–2003)
Observations (*n*)	2,053	1,963	780	311	813
Unemployment rate	−0.07 (0.01)**	0.02 (0.01)**	−0.04 (0.01)**	−0.28 (0.18)	−0.01 (0.02)
Trucking employment per square mile	0.001 (0.0003)*	0.001 (0.001)^a^	0.002 (0.001)*	0.003 (0.001)**	0.004 (0.001)**
*R*^2^	0.26	0.20	0.30	0.40	0.43

a Marginally significant (*p*= 0.09).

* *p*< 0.05.

** *p*< 0.01
